# Quantum Communication—Celebrating the Silver Jubilee of Teleportation

**DOI:** 10.3390/e22060628

**Published:** 2020-06-06

**Authors:** Rotem Liss, Tal Mor

**Affiliations:** Computer Science Department, Technion, Haifa 3200003, Israel; talmo@cs.technion.ac.il

**Keywords:** quantum communication, quantum teleportation, quantum entanglement

## 1. Introduction: Quantum Teleportation—Meaning and Influence

In 1993, Charles H. Bennett, Gilles Brassard, Claude Crépeau, Richard Jozsa, Asher Peres, and William K. Wootters published their seminal paper presenting quantum teleportation, titled “Teleporting an unknown quantum state via dual classical and Einstein–Podolsky–Rosen channels” [1]. Their paper presents and answers the question “Can we transmit an unknown quantum state *without physically sending it*?” Namely, can we send enough information about our unknown quantum state, in a way that would enable the receiver to obtain (i.e., regenerate) it? Their paper provides a striking answer: “Yes. An arbitrary state of a quantum bit (denoted by $\;|\psi \rangle ≜\cos(\frac{\theta }{2})\;|0\rangle +e^{i\phi }\sin(\frac{\theta }{2})\;|1\rangle$) can be transmitted *if* both the sender and the receiver share a maximally entangled quantum state (for example, the *singlet* state, denoted by $\;|\Psi^{-}\rangle ≜\frac{\;|01\rangle -\;|10\rangle }{\sqrt{2}}$) *and* the sender can transmit classical messages (only two standard/classical bits) to the receiver”. This answer, which presented the *quantum teleportation* protocol, has revolutionized the field of quantum communication.

Intuitively, the teleportation paper proves the equivalence “a quantum communication channel = a shared entangled state + a classical communication channel”. In particular, “*sending* an unknown state of **one** *quantum* bit can be done by *sharing* (ahead of time) **one** maximally entangled state of two *quantum* bits + *sending* **two** *classical* bits”. The above equivalence is very important, because quantum channels tend to be much less reliable (and much more prone to losses and errors) than classical channels; moreover, even if the sender and the receiver only share (many) *noisy* entangled states, they can still employ quantum teleportation by first distilling (a fewer number of) nearly *maximally* entangled states [2]. (This method, in particular, makes it possible to transmit arbitrarily faithful quantum states over a *noisy* quantum channel [2], even without using quantum error-correcting codes [3]).

**Figure 1:**
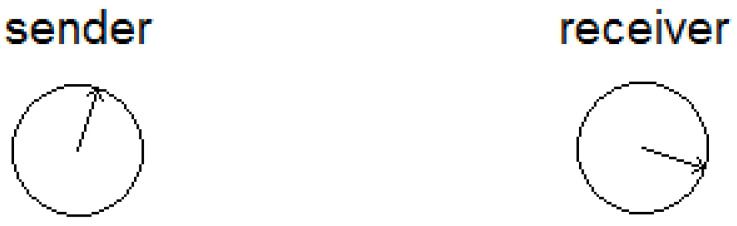
We illustrate the power of quantum teleportation by representing the quantum bit as an *arrow* (a two-dimensional arrow in this drawing; a three-dimensional arrow in general) inside a unit sphere. In the general three-dimensional case, this representation is known as the *Bloch sphere representation*. The sender would like to transmit the arrow’s direction to the receiver, without physically sending the arrow.

To see how surprising this result is, let us represent the quantum bit as an *arrow* directed at some arbitrary direction in the three-dimensional space (see Figure 1 for a two-dimensional illustration). The arrow’s direction can be represented in spherical coordinates by the two angles θ,ϕ (note that θ,ϕ are also the two angles that appear in the mathematical representation $\;|\psi \rangle ≜\cos(\frac{\theta }{2})\;|0\rangle +e^{i\phi }\sin(\frac{\theta }{2})\;|1\rangle$ of the quantum bit). Therefore, the corresponding *classical* question is “Can we transmit the arrow’s direction *without physically sending the arrow*?” The obvious classical answer is “Yes, but only if we send the real numbers θ,ϕ”. Namely, in the classical case, even when the sender *knows* the arrow’s direction, a very large number of classical bits must be sent so that the receiver can reconstruct the approximate direction of the arrow (the degree of precision dictates the number of sent bits; infinite precision requires an *infinite* number of bits). On the other hand, *two* classical bits would give us a very limited amount of information, not allowing the receiver to recover the arrow’s direction at any reasonable amount of precision. This is true even if the sender and the receiver share some information in advance, assuming that the arrow’s direction is chosen randomly and independently of the shared information. (In a limited classical case, where the sender wants the receiver to get the probability distribution of *one* biased coin, we can have some kind of “classical teleportation”, even if that distribution is unknown to the sender; see details in [4]).

The *quantum* case seems even worse: if the sender holds the unknown quantum state |ψ〉 and wants to transmit it to the receiver, the sender apparently still has to send the two real numbers θ,ϕ. Moreover, due to the peculiar properties of quantum mechanics, those real numbers are now *not even known* to the sender, because the description of $\;|\psi \rangle ≜\cos(\frac{\theta }{2})\;|0\rangle +e^{i\phi }\sin(\frac{\theta }{2})\;|1\rangle$ is unknown to the sender. (Note that the sender cannot discover the description of |ψ〉, and any attempt to do so would irreversibly damage the quantum state). Nonetheless, the quantum teleportation paper proves that by using the extraordinary power of quantum entanglement, *only two* classical bits need to be sent.

The teleportation paper is one of the most prominent examples of the counterintuitive power of quantum communication; other notable examples include quantum cryptography [5,6], violations of Bell’s inequality [7], and even the basic phenomena of quantum entanglement and EPR pairs [8].

## 2. The Discovery of Quantum Teleportation: History, Notes, and Stories

Like any groundbreaking result, there are several interesting stories surrounding the discovery of quantum teleportation. Perhaps most interesting of all is the story of the actual invention of quantum teleportation, as recounted by Gilles Brassard and printed here for the first time (except for an earlier personal account in French [9]):

“It all started in August 1992, when I was attending the annual CRYPTO conference. Charlie Bennett gave me a paper that had appeared in *Physical Review Letters* one year earlier, saying ‘I think this will interest you’. Right he was! That was the paper by Asher Peres and William (Bill) K. Wootters [10] in which they considered the following problem: if two participants hold identical copies of an unknown quantum state |ψ〉, so that the state of their joint system is ${\;|\psi \rangle }_{A}⊗\;{|\psi \rangle }_{B}$, how much information can they discover about |ψ〉 if they are restricted to local quantum operations and classical communication (this is of course what became known later as LOCC)? In that paper, Peres and Wootters studied so-called ping-pong protocols in which more information can be obtained by increasing the number of interaction rounds, but they were unable to get quite as much information as if the two identical quantum states were in the same location, enabling the possibility of a joint measurement. Their paper left open the following question: can LOCC measurements provide as much information as joint measurements?

At the time, I had never met Peres or Wootters, and in fact I had never heard of them. I met them both a few months later by a pleasant coincidence, at the October 1992 *Workshop on Physics and Computation* held in Dallas. After discussing the paper with its authors, I invited Bill to come to Montréal to give a talk about it the following month. Somehow, I had a feeling this would be momentous, and therefore I invited Claude Crépeau (who was in Paris at the time) and Charlie Bennett to attend the talk at my expense. Richard Jozsa was in the audience as well because he was my research assistant at the time. After Bill explained the conundrum, Charlie raised his hand and asked an apparently inane question: ‘What difference would it make if the two participants shared an EPR pair?’ (that’s what we called entanglement in those days). Not surprisingly, Bill replied ‘I don’t know!’ and then went on with his talk. Immediately afterwards, we all moved to my office and brainstormed about Charlie’s question. By the next morning, the answer was clear: in the presence of entanglement, one party teleports |ψ〉 to the other, who then performs the optimal joint measurement. It is fair to say that we were able to invent quantum teleportation within less than 24 hours because none of us was trying to achieve this obviously impossible task! Of course, we realized that this invention was far more important than the solution it offered to the problem at hand, but I don’t think any of us anticipated how important it would become. We quickly invited Asher to join the collaboration and, within eleven days, the paper was submitted to *Physical Review Letters*. The rest is history”.

The writing process of that seminal paper was not exempt from dilemmas. Gilles Brassard describes one of them—the length of the paper:

“Once we had a version of the paper that we really liked, we noticed that it was just a little too long for the then strict limit of four pages imposed by *Physical Review Letters*. We could not find anything that we would be comfortable leaving out. That was when a devilish idea came to me. Given that the type is smaller in figure captions (8.5 points) than in the main text (9.5 points), why not squeeze in some content there? We relegated the proof that successful teleportation of one qubit *requires* the transmission of two classical bits to what became a 27-line caption for Figure 2 (see [1]), which saved exactly the required amount of space to fit the paper snuggly in four pages. Ironically, we ended up being the first paper of its issue, and the space needed for the journal header made us spill on a fifth page!”

Another important dilemma was the order of the authors’ names. Readers unfamiliar with the advantages and disadvantages of alphabetical order may not be able to understand and appreciate the subtleties of the following story. Alphabetical order for authors’ names is customary in our field, in contrast to the “contribution order”, which is conventional in many others. There are researchers who participate only or mostly in alphabetical-order papers, and it is very important to many of them to *avoid combining the two methods*: combining in that way could have a negative potential impact on both their own research career and the careers of their alphabetic co-authors.

The original teleportation paper listed authors in alphabetical order. Charles Bennett, who frequently held the position of first author due to his last name, with many papers being cited as “Bennett et al”. felt he was being over-credited in the eyes of those accustomed to contribution order. For this reason, at some point before submission of the teleportation paper, he suggested the use of reverse alphabetical order for the authors, which would have placed Bill Wootters as first author. This idea was almost immediately rejected by Wootters himself. Gilles Brassard, who has never once strayed from alphabetical order throughout his entire career, told us years later that he felt so strongly about this issue, that he would have withdrawn his name from the paper had Bennett’s reverse authorship idea been carried out. Of course he could not have known this at the time, but taking himself off the paper might have prevented him from sharing the Wolf Prize with Charles Bennett one quarter of a century later. (Details about the Wolf Prize won by Bennett and Brassard, which they received *both* for quantum cryptography and quantum teleportation, are provided at the end of the current section).

Yet another dilemma was the *name* of the new method. Asher Peres objected to the original name “teleportation” because it mixed the Greek prefix “tele-” with the Latin-based root “port”. Peres suggested the alternative name “telepheresis”, but the other authors disagreed, so the name remained “teleportation”.

The quantum teleportation paper received excellent reviews before being accepted to *Physical Review Letters*. One of the reviewers, N. David Mermin, described the paper as a “charming, readable, thought-provoking paper”, and predicted that “this novel method […] will become an important one of the intellectual tools available to anybody […]” (see Mermin’s paper [11], where he disclosed his full referee report on the quantum teleportation paper). Finally, the paper was published on 29 March 1993 [1] in *Physical Review Letters*, profoundly advancing the field of quantum communication and bringing new researchers to the fast-evolving field of quantum information processing. In particular, it influenced Tal Mor, who is one of the authors of this editorial, as he describes below.

When the teleportation paper was published (1993), I was an M.Sc. student in Tel Aviv University (Israel) in the group led by Yakir Aharonov, together with Sandu Popescu (who was a Ph.D. student at the time) and Lev Vaidman (who was a postdoctoral researcher). All three of us (Popescu, Vaidman, and I) were extremely excited about the teleportation paper: Popescu suggested a method [12] leading to the first experimental realization of quantum teleportation [13] (note that quantum teleportation was experimentally demonstrated in 1997–1998 by three research groups [13,14,15]); Vaidman suggested teleportation of continuous quantum variables [16], leading to a theoretical extension [17] and its experimental realization [15]; and I decided to start my Ph.D. with Asher Peres, who was one of the teleportation paper’s authors. Although I concentrated on quantum cryptography, I also gave a lot of thought to quantum teleportation: I presented teleportation as a special case of POVM (generalized measurements) in my first talk at an international conference [18,19,20], and I suggested how a classical variant of teleportation could look like (a concept I published years later [4]).

The quantum teleportation paper and its experimental realizations intrigued not only scientists, but also media reporters. When one of them asked Peres whether quantum teleportation teleports only a person’s body, or also the soul, Peres answered that it teleports *only* the soul [21]—a funny, thought-provoking reply from someone like Peres, who enjoyed describing himself as a devout atheist!

During my Ph.D. and postdoctoral research, I became acquainted with all six authors of the teleportation paper. I even asked them to autograph an original reprint of the paper—so I now own the only copy of the quantum teleportation paper signed by all six co-authors! (Admittedly, it was pretty hard to obtain this signed copy. Unfortunately, Gilles Brassard, who was the last co-author to sign, lost the copy signed by the five other co-authors in his office; later, he sent me an e-mail including the “good news”—that he found the signed copy of the teleportation paper—and the “bad news”—that he lost it again; finally, he found it *again*, signed it, and immediately mailed it from Canada to me in Israel, and I received it. Then, *I* lost it in *my* office… where I may find it again some day).

Subsequently, I had two opportunities to celebrate the quantum teleportation paper and honor some of its authors at my institution (Technion, Haifa, Israel): when I organized the QUBIT 2003 conference, celebrating 10 years of quantum teleportation, with Asher Peres as the guest of honor [22]; and when I organized the QUBIT 2018 conference, celebrating the Wolf Prize of Charles Bennett and Gilles Brassard, with both of them as the keynote speakers [23].

Charles Bennett and Gilles Brassard won the 2018 Wolf Prize in physics “for founding and advancing the fields of Quantum Cryptography and Quantum Teleportation”. The jury panel acknowledged the enormous importance of quantum teleportation:

“In the 1990’s they [Bennett and Brassard], together with four colleagues, invented quantum teleportation which allows the communication of quantum information over classical channels, also a task previously believed to be impossible. Two decades after their proposal, quantum teleportation has now been demonstrated over distances exceeding 1000 kilometers and is clearly destined to play a major role in future secure communications”. [24]

## 3. The Papers in This Special Issue

This special issue is dedicated to celebrating the silver jubilee of the seminal teleportation paper, and it features contributions from various areas of quantum communication.

Francesco De Martini and Fabio Sciarrino, in their paper “Twenty years of quantum state teleportation at the Sapienza University in Rome” [25], review various experiments of quantum teleportation that were conducted at the Sapienza University in Rome, ranging from the *first* teleportation experiment (1997) to several variations and generalizations of teleportation, such as active teleportation and quantum machines based on teleportation.

Nicolas Gisin, in his paper “Entanglement 25 years after quantum teleportation: Testing joint measurements in quantum networks” [26], discusses quantum entanglement from an unusual perspective: that of entangled *measurements* rather than entangled *states*. In particular, Gisin raises the question of whether entangled measurements can be used for generating non-classical output correlations in various quantum networks, and suggests a few candidates that may present such non-classical correlations.

Gilles Brassard, Luc Devroye, and Claude Gravel, in their paper “Remote sampling with applications to general entanglement simulation” [27], provide a (classical) sampling scheme: their scheme allows the user to sample *exactly* from a discrete probability distribution when the defining parameters of this probability distribution are partitioned between several remote parties. Furthermore, they apply their sampling scheme to the classical simulation of quantum entanglement measurements in the most general scenario, and analyze its communication complexity.

William K. Wootters, in his paper “A classical interpretation of the Scrooge distribution” [28], shows how to derive a special *quantum* ensemble of pure states, known as the “Scrooge ensemble” (or “Scrooge distribution”), from a *classical* communication scenario. Specifically, he proves that a real-amplitude variant of the Scrooge distribution naturally appears in a classical communication scheme, and that the standard (complex-amplitude) Scrooge distribution appears in a modified version of the same communication scheme.

Michel Boyer, Rotem Liss, and Tal Mor, in their paper “Attacks against a simplified experimentally feasible semiquantum key distribution protocol” [29], explore the security of a semiquantum key distribution (SQKD) protocol that seems easy to implement in practice. In particular, they analyze a simplified variant of the previously published “Mirror” SQKD protocol, and prove that unlike the original Mirror protocol (which was proved completely robust), its simplified variant is completely insecure if the tolerated loss rate is high.

Kan Wang, Xu-Tao Yu, Xiao-Fei Cai, and Zai-Chen Zhang, in their paper “Probabilistic teleportation of arbitrary two-qubit quantum state via non-symmetric quantum channel” [30], propose a variant of quantum teleportation: their scheme allows teleporting an arbitrary two-qubit state from Alice to Bob, given that Alice and Bob share one partially entangled pure three-qubit state and one partially entangled pure two-qubit state. Their teleportation scheme is probabilistic and unambiguous: namely, it may fail with constant probability, but the users know whether it succeeded or failed.

We hope that the papers in this special issue give insight regarding the different areas of quantum communication—most notably, quantum teleportation, quantum entanglement, and quantum cryptography.
